# Conjugated linoleic acid metabolite impact in colorectal cancer: a potential microbiome-based precision nutrition approach

**DOI:** 10.1093/nutrit/nuae046

**Published:** 2024-05-10

**Authors:** Adriana González, Asier Fullaondo, Javier Rodríguez, Cristina Tirnauca, Iñaki Odriozola, Adrian Odriozola

**Affiliations:** Department of Genetics, Physical Anthropology and Animal Physiology, University of the Basque Country UPV/EHU, Bilbao, Spain; Department of Genetics, Physical Anthropology and Animal Physiology, University of the Basque Country UPV/EHU, Bilbao, Spain; Department of Oncology, Clínica Universidad de Navarra, Pamplona, Spain; Departamento de Matemáticas, Estadística y Computación, Universidad de Cantabria, Santander, Spain; Health Department of Basque Government, Gipuzkoa, Donostia-San Sebastián, Spain; Department of Genetics, Physical Anthropology and Animal Physiology, University of the Basque Country UPV/EHU, Bilbao, Spain

**Keywords:** anticarcinogen, colorectal cancer, conjugated linoleic acid, gut microbiota, precision nutrition

## Abstract

Colorectal cancer (CRC) is the second most deadly and the third most diagnosed cancer in both sexes worldwide. CRC pathogenesis is associated with risk factors such as genetics, alcohol, smoking, sedentariness, obesity, unbalanced diets, and gut microbiota dysbiosis. The gut microbiota is the microbial community living in symbiosis in the intestine, in a dynamic balance vital for health. Increasing evidence underscores the influence of specific gut microbiota bacterial species on CRC incidence and pathogenesis. In this regard, conjugated linoleic acid (CLA) metabolites produced by certain gut microbiota have demonstrated an anticarcinogenic effect in CRC, influencing pathways for inflammation, proliferation, and apoptosis. CLA production occurs naturally in the rumen, and human bioavailability is through the consumption of food derived from ruminants. In recent years, biotechnological attempts to increase CLA bioavailability in humans have been unfruitful. Therefore, the conversion of essential dietary linoleic acid to CLA metabolite by specific intestinal bacteria has become a promising process. This article reviews the evidence regarding CLA and CLA-producing bacteria as therapeutic agents against CRC and investigates the best strategy for increasing the yield and bioavailability of CLA. Given the potential and limitations of the present strategies, a new microbiome-based precision nutrition approach based on endogenous CLA production by human gut bacteria is proposed. A literature search in the PubMed and PubMed Central databases identified 794 papers on human gut bacteria associated with CLA production. Of these, 51 studies exploring association consistency were selected. After excluding 19 papers, due to health concerns or discrepancies between studies, 32 papers were selected for analysis, encompassing data for 38 CLA-producing bacteria, such as Bifidobacterium and Lactobacillus species. The information was analyzed by a bioinformatics food recommendation system patented by our research group, Phymofood (EP22382095). This paper presents a new microbiome-based precision nutrition approach targeting CLA-producing gut bacterial species to maximize the anticarcinogenic effect of CLA in CRC.

## INTRODUCTION

Colorectal cancer (CRC) is a significant worldwide health burden.^1^ Unfortunately, despite improvements in prevention, diagnosis, and treatment, CRC is the second deadliest cancer for both sexes globally and the third most commonly diagnosed (Globocan, 2020; https://gco.iarc.fr/). Therefore, researchers worldwide are focused on designing new strategies for managing CRC.^2^

The etiology of CRC is similar to that of many complex diseases affected by genetic and environmental factors.^3^ According to the scientific literature, inherited genetics contributes to CRC in 12%–35% of cases,^4^^,^^5^ while 60%–65% of CRC cases are sporadic.^6^ Environmental factors such as alcohol, tobacco, obesity, physical inactivity, and unbalanced diets contribute to this sporadic causality.^7^ The relationship between diet and CRC has been solidly established, and it has been reported that CRC incidence could be reduced by 70% following healthy and balanced nutrition.^8^^,^^9^

Scientific research has suggested that the gut microbiota could be “the missing link” between nutrition and the incidence of CRC.^10–13^ Gut microbiota is the diverse community of microorganisms living in the host gut in symbiotic relationship.^14^ Intestinal bacteria perform essential functions in human health^15^ and performance,^16^^,^^17^ such as producing essential metabolites and regulating the digestion and absorption of dietary fiber, the immune system, and intestinal and systemic inflammation. Increasing scientific evidence shows the fundamental role the intestinal microbiota plays in the initiation, development, and metastasis of CRC.^18–20^ The microbiota is capable of being modulated, unlike our genome, which remains more constant throughout our lives.

The main modulating factors of the intestinal microbiome are nutrition, physical activity, circadian rhythms, and exposure to xenobiotics and antibiotics.^21^ Therefore, lifestyle interventions, with nutrition as the main factor, contribute to rebalancing the intestinal microbiota. Changes in the diet lead to changes in the quantitative and qualitative microbiota composition, which affect health.^2^ Consumption of diets high in saturated fats, red meat, highly processed foods, and sugars can generate imbalances in the intestinal microbiota that cause inflammation, a main triggering factor in 20%–30% of CRC cases.^22–24^ In addition, the risk of CRC increases with high fat and red meat intake, since the intestinal microbiota can use them to generate carcinogenic metabolites, such as N-nitroso compounds.^25–27^ Scientific evidence has demonstrated the ability of the gut microbiota to synthesize and regulate metabolites strongly related to CRC.^28^^,^^29^ Furthermore, the ability of the gut microbiota to produce these metabolites has been shown to vary in response to diet. Apart from N-nitroso compounds, short-chain fatty acids, bile acids, and conjugated linoleic acid (CLA) are important metabolites produced by gut microbiota that are associated with CRC.^30^

CLA is considered a fatty acid with health-promoting effects, and its anticancer properties in vivo and in vitro have been broadly studied and recognized.^29^^,^^31^ Specifically, the anticarcinogenic effect of CLA in CRC has been reported as occurring through various complex mechanisms, influencing inflammation, proliferation, and apoptosis pathways.^2^ CLA is produced mainly by rumen bacteria, such as *Butyrivibrio fibrisolvens,*^32^ and human bioavailability is limited to consuming products from ruminants.^33^^,^^34^

In recent years, several attempts have been made to develop various strategies to increase the amount and bioavailability of intestinal CLA. Those strategies have included CLA supplements to microbiome-based precision nutrition, such as linoleic acid (LA) intake as a substrate for CLA production by microbiota; functional food; and genetically modified bacteria.

In this review, we have discussed the current scientific evidence related to CLA and CLA-producing bacteria as a therapeutic against CRC, and investigated the best strategy for increasing the CLA amount and bioavailability. We have reviewed the current strategies, discussing the potential and limitations of those strategies. A next-generation microbiome-based precision nutrition approach has also been proposed, focusing on the growth of specific CLA-producing bacteria from human gut microbiota to maximize its anticarcinogenic effect in CRC.

## MATERIALS AND METHODS

### Literature review

We carried out a literature search in the Pubmed and Pubmed Central Repositories of the National Center of Biotechnology Institute for: “colorectal cancer” combined with the terms “conjugated linoleic acid,” “bacteria,” “gut,” or “precision nutrition,” in articles prior to and including February 2023, and obtained 2328 articles. From these, 794 articles relating to human gut commensal bacteria and CLA production were selected, in an endeavor to identify the most significant bacteria at genus and species level associated with CLA production in the human gut microbiota. From these, 51 articles that discussed bacteria consistently associated with CLA production were selected. Reading within this preliminary selection, those bacteria previously associated with detrimental health effects were identified, and the corresponding articles were discarded. Finally, 32 articles were included in the review. Data within these articles on CLA-producing gut commensal bacteria in the CRC context were used to construct Table 1.

**Table 1:** Specific CLA isomers, CLA precursors, and CLA products generated by each bacterial species, with reference sources

Bacterial species	CLA isomers				CLA precursors			CLA products
	c9, t11-CLA	t10, c12-CLA	t9, t11-CLA	t9, c11-CLA	HFA	HY2	HY1	VA
*Bifidobacterium adolescentis*	^70^ ^,^ ^105^	^70^	^70^		^101^			
*Bifidobacterium animalis* subsp. *lactis*	^69^ ^,^ ^105^					^69^		
*Bifidobacterium bifidum*	^70^ ^,^ ^105–107^		^106^					
*Bifidobacterium breve*	^70^ ^,^ ^86^ ^,^ ^101^ ^,^ ^105^ ^,^ ^106^ ^,^ ^108^ ^,^ ^109^	^86^ ^,^ ^109^	^70^ ^,^ ^101^ ^,^ ^105^ ^,^ ^106^ ^,^ ^109^					
*Bifidobacterium catenulatum*	^105^							
*Bifidobacterium dentium*	^70^ ^,^ ^105^	^70^	^70^					
*Bifidobacterium longum*	^86^ ^,^ ^105^	^86^						
*Bifidobacterium pseudocatenulatum*	^70^ ^,^ ^105^ ^,^ ^110^	^70^	^70^					
*Bifidobacterium pseudolongum*	^105^ ^,^ ^106^		^106^					
*Butyrivibrio fibrisolvens*	^32^ ^,^ ^111^ ^,^ ^112^							^101^ ^,^ ^127^
*Enterococcus faecium*	^113^							
*Eubacterium siraeum*					^101^			
*Faecalibacterium prausnitzii*					^101^			
*Lactobacillus acidophilus*	^69^ ^,^ ^86^ ^,^ ^102^ ^,^ ^103^ ^,^ ^114^ ^,^ ^115^	^69^ ^,^ ^86^ ^,^ ^114^ ^,^ ^115^	^69^ ^,^ ^102^ ^,^ ^103^ ^,^ ^114^			^102^ ^,^ ^103^	^102^ ^,^ ^103^	
*Lactobacillus brevis (Levilactobacillus brevis)*	^69^ ^,^ ^103^	^69^	^69^ ^,^ ^103^			^103^ ^,^ ^104^		
*Lactobacillus casei (Lacticaseibacillus casei)*	^69^ ^,^ ^86^ ^,^ ^114^	^69^ ^,^ ^86^ ^,^ ^114^	^69^ ^,^ ^114^					
*Lactobacillus crispatus*	^69^	^69^	^69^					
*Lactobacillus delbrueckii* subsp. *bulgaricus*	^69^ ^,^ ^86^	^69^ ^,^ ^86^	^69^					
*Lactobacillus fermentum (Limosilactobacillus fermentum)*	^116^	^116^	^116^					
*Lactobacillus gasseri*	^69^	^69^	^69^					
*Lactobacillus helveticus*	^69^	^69^	^69^					
*Lactobacillus paracasei (Lacticaseibacillus paracasei)*	^103^ ^,^ ^104^		^103^ ^,^ ^104^			^103^ ^,^ ^104^	^103^ ^,^ ^104^	
*Lactobacillus pentosus (Lactiplantibacillus pentosus)*	^104^		^104^			^103^ ^,^ ^104^	^103^ ^,^ ^104^	
*Lactobacillus plantarum (Lactiplantibacillus plantarum)*	^69^ ^,^ ^82^ ^,^ ^86^ ^,^ ^103^ ^,^ ^104^	^86^	^69^ ^,^ ^82^ ^,^ ^103^			^69^ ^,^ ^103^	^103^	
*Lactobacillus reuteri (Limosilactobacillus reuteri)*	^69^ ^,^ ^70^ ^,^ ^117^	^117^			^101^			
*Lactobacillus rhamnosus (Lacticaseibacillus rhamnosus)*	^69^ ^,^ ^103^ ^,^ ^118^	^69^ ^,^ ^118^	^69^ ^,^ ^103^			^103^	^103^	
*Lactobacillus salivarius (Ligilactobacillus salivarius)*		^119^						
*Lactococcus lactis*		^120^			^101^			
*Leuconostoc mesenteroides*	^121^ (NS)							
*Pediococcus pentosaceus*	^122^							
*Propionibacterium acidipropionici (Acidipropionibacterium acidipropionici)*	^123^							
*Propionibacterium freudenreichii*	^70^ ^,^ ^101^ ^,^ ^123–125^	^101^	^101^	^124^ ^,^ ^125^				
*Propionibacterium thoenii (Acidipropionibacterium thoenii)*					^101^			
*Roseburia faecis*					^101^			
*Roseburia hominis*								^101^ ^,^ ^127^ ^,^ ^128^
*Roseburia intestinalis*					^101^			
*Roseburia inulinivorans*								^101^ ^,^ ^127^ ^,^ ^128^
*Streptococcus thermophilus*	^86^	^86^						

Taxonomic names in parentheses refer to the current names in the National Center of Biotechnology Institute Taxonomy Browser (https://www.ncbi.nlm.nih.gov/Taxonomy/Browser/wwwtax.cgi); blank cells indicate that, within the authors’ knowledge, that bacterium has not been reported to produce a specific metabolite. *Abbreviations*: c9, t11-CLA, *cis*-9, *trans*-11-CLA; CLA, conjugated linoleic acid; HFA, hydroxy fatty acids; HY1, 10-hydroxy-*trans*-octadecenoic acid; HY2, 10-hydroxy-*cis*-12-octadecenoic acid; NS, did not specify the CLA isomer produced; t9, t11-CLA, *trans*-9, *trans*-11-CLA; t9, c11-CLA, *trans*-9, *cis*-11-CLA; t10, c12-CLA, *trans*-10, *cis*-12-CLA; VA, vaccenic acid.

## CLA AS A THERAPEUTIC AGENT AGAINST CRC

Scientific evidence suggests that CLA could be a potential therapeutic against CRC. From the pleiotropic nature of CLA’s metabolic effects, it is thought that the different CLA isomers may act as competitive ligands for various signaling pathways.^35–37^ Studies have shown that CLA isomers exert different biological activities.^38^ Currently, the isomers *trans*-10, *cis*-12 (t10, c12/10E, 12Z)-CLA and *cis*-9, *trans-*11 (c9, t11/9Z, 11E)-CLA are the most studied.^39^ In the case of colorectal carcinogenesis, researchers have shown that both t10, c12-CLA^40^ and the c9, t11-CLA^41^^,^^42^ exert anticancer effects. However, the t10, c12-CLA isomer is considered more potent than the c9, t11-CLA isomer, because some studies have shown that t10, c12-CLA but not c9, t11-CLA exerts anticancer effects in human colon cancer cells.^43–45^

From the dietary point of view, a negative correlation has been shown between the consumption of CLA-containing dairy food and the incidence of CRC.^46^ CLA mixture supplementation has also been associated with a reduction in the serum levels of tumor necrosis factor (TNF)-α, interleukin (IL)-1β, C-reactive protein, matrix metallopeptidase (MMP)-9, and MMP-2, suggesting that CLA may reduce angiogenesis, tumor invasion, and inflammation in rectal cancer patients undergoing chemoradiotherapy.^47^ In addition, a lower t10, c12-CLA content in the feces of patients with colorectal adenomatous polyps has been detected compared with healthy subjects.^29^

Furthermore, animal models and human cell in vitro studies have reported that CLA stimulates apoptosis,^48–51^ inhibits growth and proliferation,^52–56^ and alters the eicosanoid synthesis of colon cancer cells.^49^^,^^51^ CLA colon carcinogenesis inhibition may be mediated by its ability to suppress Bcl-2 proteins and to increase Bax,^49^ caspase 3, and caspase 9 apoptosis-related proteins.^48^^,^^57^ It has also been shown that CLA may exert its anticarcinogenic activity, changing arachidonic acid metabolism^58^^,^^59^ and downregulating insulin like growth factor 1 receptor (IGF-IR), phosphatidylinositol 3-kinase/AKT serine/threonine kinase 1 (PI3K/Akt), extracellular signal-regulated kinase (ERK)-1/2,^60^ adenomatous polyposis coli (APC)-β-catenin- T-cell factor 4 (TCF-4), and peroxisome proliferator-activated receptor (PPAR) δ signaling pathways.^41^ In addition, CLA could exert an action in the cell cycle control, diminishing differentiation and proliferation through p21 induction by p53-dependent and -independent mechanisms, resulting in negative regulation of cyclin dependent kinase (CDK)/cyclins and proliferating cell nuclear antigen (PCNA) growth activities.^54^^,^^61^

Although most studies showed an anticarcinogenic effect of CLA in CRC, in some articles, no CLA effect in inhibiting colon carcinogenesis in rats was reported,^62^^,^^63^ and in a few cases, colon carcinogenesis progression in vitro was even described.^64^^,^^65^ These conflicting results could be clarified through research into the mechanisms of bioactive CLA in colorectal tumors, specifically in vivo experiments and clinical trials.^39^^,^^43^

## STRATEGIES FOR INCREASING THE CLA AMOUNT AND BIOAVAILABILITY

CLA is the term used for a group of positional and geometric double-bond isomers of LA,^66^^,^^67^ c9, t11-CLA being the major isomer, and t10, c12-CLA one of the minor isomers.^41^ CLA is produced as an intermediate product in LA (C18:2) to stearic acid (C18:0) biohydrogenation, which is mainly carried out by bacteria in the rumen of herbivores.^33^ LA can be converted to CLA directly or through the formation of hydroxy fatty acid (HFA) intermediates, such as 10-hydroxy-*cis* octadecenoic acid (HY2) and 10-hydroxy-*trans* octadecenoic acid (HY1).^68^^,^^69^ The isomer c9, t11-CLA can be absorbed by the intestinal epithelial cells or be further hydrogenated by bacteria to *trans*-11 octadecenoic acid (vaccenic acid [VA]),^66^ which can be reduced to stearic acid.^70^ In tissue, the isomer c9, t11-CLA can be produced in lower amounts using VA as substrate.^70^ The bacterial hydrogenation of isomer t10, c12-CLA produces c6, t10-C18:2 instead of VA, which is also converted to stearic acid.^33^ As a result of this process, CLA is accumulated as a minor component in the milk and tissue fat of ruminants and ranges from 2.9 mg CLA/g fat to 7.1 mg CLA/g fat,^71^ an amount influenced mainly by the feeding and breeding regimen.^72^

In humans, CLA bioavailability has been attributed directly to the consumption of ruminant-derived products in which c9*,* t11-CLA is the dominant isomer,^33^^,^^34^ such as lamb or beef meat, milk, cheese, yogurt, butter, and other dairy products. Notably, milk is the ruminant-derived product with the highest CLA content, having almost 6 times more CLA than the meat with the highest content.^73^ Unfortunately, the amounts of CLA in these products are insufficient to reach the dose indicated as therapeutic in humans (3 g/day–6 g/day).^74^

Due to the low CLA bioavailability in humans, strategies involving the intake of CLA directly as a supplement have been explored. In 2010, CLA combination (c9, t11, and t10, c12-CLA) was recognized as a safe food ingredient in amounts up to 3.5 g/day for 6 months by the European Food Safety Authority.^75^ It should be emphasized that most of the current CLA supplements are obtained through the chemical isomerization of LA by catalysis, which can lead to the production of harmful metabolites, such as RuCl_3_, associated with skin corrosion and toxic pneumonitis,^76^ as well as the production of unwanted hydrogenated by-products such as stearic and oleic acids.^77–79^ Commercial supplements mainly comprise a mixture of the c9, t11, and t10, c12-CLA isomers,^80^^,^^81^ instead of a single isomer. Moreover, commercial supplements usually contain additional compounds, such as linoleic, oleic, palmitic, and stearic acids.^81^

The finding that some commensal bacteria in the human gut microbiota can produce CLA has received considerable attention from researchers.^33^^,^^38^ As a result, various microbiome-based precision nutrition approaches have been developed, seeing to increase the amount of CLA available in humans. These strategies range from increasing LA intake^82^ to the development of functional foods with CLA-producing bacteria^33^ or CLA-producing genetically modified microorganisms.^77^ In the following sections, the potential and limitations of each strategy are discussed in detail, in order to shed light on what could be the best strategy for increasing the CLA amount and bioavailability in the CRC context.

## CURRENT MICROBIOME-BASED PRECISION NUTRITION APPROACHES

### LA intake-based strategy

Most studies use free LA^82^ and oils or foods rich in LA^83^ to test the ability of various bacteria to produce CLA. However, increasing LA intake to improve the CLA amount and bioavailability has important drawbacks.

First, increased n-6 fatty acids intake, such as intake of LA, has been associated with chemically induced carcinogenic effects^84^^,^^85^ and, therefore, is not currently recommended.^86^

Second, previous studies have estimated that LA is already present in humans, because LA excretion of approximately 340 mg of LA/day has been reported.^87^ The available LA varies according to the amount ingested and the amount that has been absorbed in the small intestine.^86^

Third, an increase in LA consumption has not been associated with an increase in CLA production. In the study by Taylor et al (2020), the fecal recovery of CLA in 115 subjects with similar high-quality dietary intake patterns did not correlate with their daily dietary intake of LA or CLA.^88^ The participants were grouped into consumers and non-consumers of fermented foods. As expected, the consumers of fermented foods were found to have a larger amount of CLA and CLA-producing bacteria, several of which are associated with fermented food. The non-consumers subgroup were found to have other bacteria related to CLA synthesis.^88^ Therefore, the main conclusion was that the increased amount of CLA present in consumers of fermented foods could not be entirely explained by fermented food–associated bacteria, and there had to be other determinants.^88^ The growth of CLA-producing bacteria in non-consumers of fermented foods could be related to other dietary components. Future studies could be needed to test this hypothesis.

Based on these findings, the increase in LA intake does not seem to be a primary strategy for increasing the CLA amount and its bioavailability.

### Functional food and genetically modified bacteria

Foods that naturally contain CLA usually have an insufficient amount to exert therapeutic effects, so various strategies have been developed to produce CLA-enriched functional foods.^73^

Functional foods are those with a traditional appearance, included in a daily diet, containing beneficial additives that provide health-related benefits.^89^ Functional foods, such as cheese and yogurt enriched with CLA-producing bacteria, have been developed, with promising short-term results.^33^ For example, after feeding mice with functional cheeses, a significant change in their fatty acid composition was shown at the tissue level (an increase in CLA content of 2-fold in the liver and 3-fold in the intestine), and a protective effect on the viability of intestinal cells was reported after treatment with the oxidant agent 1,2-dimethylhydrazine.^90^ Likewise, advances in the description of the synthesis of CLA have allowed the design of genetically modified recombineered bacteria, such as *Escherichia coli*^91^ and *Yarrowia lipolytica,*^92^ super-producers of CLA, with the idea of using them as bacterial factories, for the commercial production of probiotics^93^ or CLA supplements.^77^

The direct dietary intake of CLA from CLA-enriched functional food has the disadvantage of having to pass through the entire gastrointestinal tract.^94^ Nanocarriers have been developed for producing functional food, and through these hydrophobic compounds can be efficiently and stably delivered.^95^ CLA loaded in lipid-based nanoparticles has been studied as a potential approach for fortifying low-fat milk,^95^ but studies with in vivo animals and humans are needed to assess its viability in real food applications.^96^

In addition, although some CLA-producing bacteria can be consumed in food or as probiotic supplements, they usually are not maintained in the intestine; therefore, the increase in CLA production only lasts while the consumption is maintained.^94^

## NEXT-GENERATION MICROBIOME-BASED PRECISION NUTRITION APPROACH

Despite the advances mentioned above regarding strategies based on CLA supplements and current microbiome-based precision nutrition approaches, the future therapeutic use of CLA against CRC requires the development of new strategies. Assuming that a balanced diet is preferable over dietary supplementation in promote health,^97^ these strategies should focus on increasing the bioavailable CLA from the intake of natural food as a part of the diet, to achieve long-lasting CLA bioavailability, while maintaining an environmentally friendly approach.^73^

In accordance with that philosophy, next-generation microbiome-based precision nutrition approaches are being developed, exploring comprehensive and dynamic nutritional recommendations based on individual variables such as microbiome, health status, and dietary patterns.^98^ However, to the best of our knowledge, these approaches have not been previously focused on the CLA and CRC context, despite the scientific evidence in the literature regarding CLA’s potential as a therapeutic agent against CRC through a CLA-producing human gut microbiota.

Therefore, in this section, we explore the potential of a new microbiome-based precision nutrition approach based on the endogenous production of CLA by human gut bacteria.

The development of this new approach requires two main steps: first, a review of the scientific evidence for the relationship between human gut microbiota and the target phenotype; second, the creation of algorithms and bioinformatics tools for handling the significant scientific and computational challenges. These challenges relate to the high number of variables and their interactions, similar to those involved in cancer disease prediction and classification.^99^ In this context, our group has developed the Phymofood bioinformatics tool (patent number P22382095) to promote the optimal growth of selected target bacteria in the human gut microbiota by individualizing the dietary intake of prebiotics (bacterial food) and diet components (food).

In the following sections, we discuss the selection of target CLA-producing human gut bacteria, discarding bacteria only inconsistently associated with CLA and those with detrimental health effects. Finally, based on this knowledge, we propose a new microbiome-based precision nutrition approach, applying the Phymofood bioinformatics tool (patent P22382095) to identify foods that can promote the optimal growth of the selected target CLA-producing bacteria.

### Target bacteria selection: diversity in CLA isomers, pathways, and synthesis

Target bacteria selection is critical in developing a next-generation microbiome-based precision nutrition strategy. In the present review, after identifying 55 CLA-producing bacteria, only 38 were selected. The selection criteria are discussed below, but mainly related to the bacteria’s ability to produce the various CLA isomers (mainly c9, t11, and/or t10, c12-CLA), CLA precursors (HFA), or CLA products (VA).

CLA isomers may act as ligands that compete for different signaling pathways.^35–37^ The anticarcinogenic properties of CLA are mainly attributed to the c9, t11, or t10, c12-CLA isomers.^41^^,^^100^ Of the 38 bacteria, 31 were included because of their ability to produce one or both of the isomers (Table 1).^32^^,^^69^^,^^70^^,^^82^^,^^86^^,^^101–125^ Likewise, c9, t11, and t10, c12-CLA mixtures with other isomers,^61^ and undetermined CLA isomeric mixtures,^60^ have also shown anticarcinogenic effects against CRC. For this reason, *trans-9, trans 11-* (t9, t11)-CLA, and *trans-9, cis-11* (t9, c11)-CLA isomers have also been included in Table 1.^69^^,^^70^^,^^82^^,^^101–106^^,^^109^^,^^114^^,^^116^^,^^124^^,^^125^

One of the main CLA production pathways in the human gut microbiota is not directly from LA to the various CLA isomers. Instead, it seems to be via HFA intermediates such as HY2 and HY1. Possibly, the HFA produced by one bacterium could be used as a substrate for the production of CLA by another bacterium. These interactions are feasible but unknown. Because of that, if any of the 31 selected bacteria produce CLA through these intermediates, it is indicated in Table 1^69^^,^^101–104^ (as is the case for *Lactobacillus acidophilus*, for example).

Moreover, 5 bacterial taxa that produce HFA, but not CLA, have been included in Table 1: *Eubacterium siraeum*, *Faecalibacterium prausnitzi*, *Propionibacterium thoenii*, *Roseburia faecis*, and *R. intestinalis*.^101^ It is possible that *L. acidophilus,* for example, could take advantage of the HFA produced by these 5 bacterial taxa. To illustrate these known bacterial interactions, we could use the following example. Three of the selected bacteria are propionibacteria, and the increase in their relative abundance is usually associated with the consumption of probiotic foods that contain them, as in the case of Swiss-type cheese.^126^ Recent research has shown that some species of *Propionibacterium*, such as *P. acidipropionici*, can use galactooligosaccharides and lactulose as prebiotics and metabolize them to oligosaccharides, which in turn can be used as prebiotics for other bacterial species.^126^

Finally, the c9, t11-CLA isomer can be produced using VA as a substrate in the tissue through the Δ9-desaturase pathway,^70^ although the contribution of this pathway to the CLA pool is minimal. Bacteria capable of producing VA that could increase the pool of this product in the tissues were included in Table 1; we note whether the 36 already selected bacteria, such as *Butyrivibrio fibrisolvens*,^101^^,^^127^ also produce VA, and 2 more bacterial species were included because they stood out for producing VA: *R. hominis* and *R. inulinivorans*.^101^^,^^127^^,^^128^

It is essential to highlight that advances in the area foreseeably will take place in the coming years. Therefore, the list in Table 1 remains open to incorporating new bacteria from the intestinal microbiota capable of producing CLA.

### Target bacteria discard

Of the CLA-producing bacteria, 17 were not included in the final list of target bacteria because of CLA-production inconsistencies or detrimental health effects being associated with them.

Fourteen of these 17 bacteria were not included due to issues related to their ability to synthesize CLA. *Anaerostipes caccae, Eubacterium hallii, Eubacterium rectale, Eubacterium ventriosum,* and *Propionibacterium jensenii* have low percentages of LA metabolization and there has been no detection of the formation of any CLA isomer.^101^  *Anaerostipes hadrus* and *Eubacterium eligens* were included as CLA producers in the study by Taylor et al (2020),^88^ but no other reference source was found, so they were discarded. *Bifidobacterium angulatum* and *Bifidobacterium infantis* have been described as producers of the c9, t11-CLA isomer, but in low or negligible amounts.^70^^,^^105^  *B. infantis* has also been reported as a producer of the c9, t11-CLA isomer, and, to a lesser extent, of t10, c12-CLA,^86^ and HFA,^101^ so it was discarded due to the inconsistencies. Within the genus *Butyrivibrio*, the species *B. fibrisolvens* is the most efficient producer of CLA; it was decided not to include the species *B. proteoclasticus*, since it produces 3.5 times less CLA than *B. fibrisolvens*.^129^  *Lactobacillus curvatus* and *Lactobacillus sakei* strains were discarded because of their minimal conversion of LA into CLA (between 2% and 5%).^130^ Although, to the authors’ knowledge, there is no standard consensus, a conversion rate in that range is usually considered low within the specialized literature.^70^^,^^101^^,^^130^^,^^131^ While LA isomerases have been described in *Rhodococcus erythropolis*, it was not included because no CLA production was detected.^132^ Some strains of *Megasphaera elsdenii* have been reported as producing t10, c12-CLA,^133^ but these results have been questioned. *M. elsdenii* was not included because researchers did not detect this product formation^134^ in a subsequent study. However, we leave the possibility open for reselecting these bacteria in the future.

Three of the 17 CLA-producing bacteria were not included due to their association with detrimental health effects. First, although *Clostridium sporogenes* is a producer of c9, t11-CLA, and other CLA isomers,^135^ it is also a spore-forming gram-positive bacterium,^136^ which is considered a rare clinical pathogen associated with septicemia^137^^,^^138^ and bacteremia in an immunocompetent patient.^139^ Second, the LA metabolizer and HFA producer, *Eubacterium ruminantium*,^101^ was discarded because it has been referred to as the single standard adenoma-associated marker in CRC.^140^ Finally, although *Propionibacterium acnes* produces t10, c12-CLA,^141–143^ it has been discarded due to its association with acne pathogenesis,^144^ infections of medical devices,^145^ and prostate cancer.^146^

### Target CLA-producing gut commensal bacteria in CRC

To select target CLA-producing bacteria, 55 were preliminarily selected, on the basis of significant association with CLA production. However, 17 of those bacteria were discarded, due to inconsistencies in the findings reported in the scientific literature, or because they were associated with detrimental health effects in other research.

Finally, 38 bacteria were selected and grouped in Table 1 according to the available scientific evidence concerning their relationship with CLA production.

Table 1 includes details of the specific CLA isomers (c9, t11-; t10, c12-; t9, t11-; and t9, c11-CLA), CLA precursors (HFA such as HY2 and HY1), and CLA products (VA) that are produced by each target bacteria.

### CLA as a potential link between probiotics and anticarcinogenic effect

Research on probiotics as potential agents for managing CRC is becoming increasingly important.^2^^,^^94^ The use of probiotics in several clinical trials has demonstrated an ameliorating effect on chemotherapy side effects, such as reduction of severe diarrhea and abdominal discomfort,^147^ and of infections in the context of CRC.^148^ Also, probiotics such as *P. freudenreichii* combined with TRAIL-based CRC therapy have been proposed, since they can increase its tolerance and efficacy.^149^ Therefore, attempts are being made to describe the pathways by which probiotics exert their anticarcinogenic effect on colorectal epithelial cells, such as the direct production of anticancer compounds, the degradation or inhibition of the synthesis of carcinogenic compounds, and the induction of proapoptotic and antiproliferative effects.^2^

Some anticancer pathways of prebiotics in which the effector molecule is unknown are also action pathways of CLA. In relation to the target bacteria selected in this review, this is the case for some of the antiproliferative and proapoptotic effects exerted by *L. casei*^150^ and *L. rhamnosus*.^24^ Therefore, CLA may also be, at least in part, one of the links between probiotics and their ability to exert anticancer effects, as other authors have already begun to suggest.^2^^,^^94^ It is noteworthy that 25 of the 38 CLA-producing target bacteria listed in Table 1 are considered probiotics and are included in the revised list of microorganisms with Qualified Presumption of Safety (QPS)—microorganisms recommended for safety risk assessments to be carried out by the European Food Safety Authority (https://www.efsa.europa.eu/en/topics/topic/qualified-presumption-safety-qps). The target bacteria included in the European Food Safety Authority list are *B. adolescentis*, *B. animalis*, *B. bifidum*, *B. breve*, *B. longum*, *L. acidophilus*, *L. brevis*, *L. casei*, *L. crispatus*, *L. delbrueckii* subsp. *bulgaricus*, *L. fermentum*, *L. gasseri*, *L. helveticus*, *L. paracasei*, *L. pentosus*, *L. plantarum*, *L. reuteri*, *L. rhamnosus*, *L. salivarius*, *L. lactis*, *L. mesenteroides*, *P. pentosaceus*, *P. acidipropionici*, *P. freudenreichii*, and *S. thermophilus.*

### Food recommender system application to target CLA-producing bacteria

The selection of the target CLA-producing bacteria (Table 1) is a valuable contribution as a scientific base for the future practical application of microbiome-based precision nutrition in the context of CRC. Based on the knowledge reviewed and discussed in the present review, a bioinformatics tool such as the Phymofood food recommender system (patent P22382095) can be used to individualize nutrition to promote the optimal growth of target CLA-producing bacteria in human gut microbiota. Although the algorithms and procedure details are described in patent P22382095, the main features and steps in the system are explained below. The process consists of selecting prebiotics and related food that theoretically favor the growth of target CLA-producing bacteria.

First, the contribution of each food to promoting the growth of each target bacterium is estimated on a scale of 0 to 1. The maximum value (1) is given if there exists direct scientific evidence of an association between that particular food and the growth target bacterium. If there is no known direct association, values are assigned to each food depending on whether the prebiotics contained in the food are target-bacterium prebiotics or not. In this case, a maximum value (1) is assigned to a food if all the prebiotics contained in the food have been reported as target-bacterium prebiotics, a minimum value (0) is given if none of them are, and an intermediate value based on a harmonic series is given if only some prebiotics contained in the food are target-bacterium prebiotics. Then, a vector that records all target-bacteria values is created for each food, where each vector element is the value assigned previously. These vectors are used to create a ranking in which the first food is the one that most contributes (the sum of all values is the highest) to the growth of target CLA-producing bacteria.

A set of 99 foods and 18 prebiotics were included in this study. The set of prebiotics considered included xylooligosaccharides, resistant starch, fructooligosaccharides, inulin, pectin, resveratrol, quercetin, raffinose, arabinoxylan-oligosaccharides, arabinogalactans, galactooligosaccharides, beta-glucans, lignans, ellagitannins, curcumin, anthocyanins, and isoflavones.

**Figure 1:**
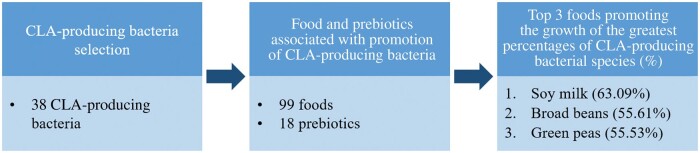
Flow diagram illustrating the steps carried out in the application of a food recommender system application to target CLA-producing bacteria.

Noting the prebiotics for each of the 38 target CLA-producing bacteria, and the presence of each prebiotic in each food, yielded the top 3 most-complete foods for this target set of CLA-producing bacteria: soy milk, promoting the growth of 63.09% of the whole set of target bacteria, broad beans (55.61%), and green peas (55.53%) (Figure 1).

This kind of microbiome-based precision nutrition approach allows healthcare professionals and researchers to identify individual target foods, while considering the additional specific requirements of each person, such as food allergies, and incompatibilities between specific foods, particular medications, or treatments.

Thus, careful selection of target CLA-producing bacteria, considering the current scientific evidence, and posterior application of a food recommender system constitutes a valuable tool for theoretically maximizing the optimization of an individual’s gut microbiota. The main potential advantages of this kind of strategy are that it is based on natural food intake as a part of a balanced diet, and th ebioactive metabolites are produced directly in the human gut. Moreover, following an individualized diet could allow these bacteria to colonize and maintain themselves in the intestine. To date, Phymofood has been tested in longitudinal case studies to promote the abundance of probiotic bacteria in healthy subjects, as detailed in patent P22382095. Consequently, future longitudinal interventions based on this approach could lead to a long-lasting anticarcinogenic effect from CLA produced by target bacteria in both healthy individuals and CRC patients.

Health recommendations advocate precision nutrition interventions targeting individual needs. Promising results have already been obtained with CLA supplementation and CLA loading in nanoparticles in functional foods. These strategies could complement next-generation microbiome-based precision nutrition to improve the amount and bioavailability of CLA in both healthy individuals and patients with CRC.

## FUTURE PERSPECTIVES

In future research, these microbiota precision nutrition approaches need to be tested in a longitudinal study in which various individuals would be analyzed before and after a precision nutrition intervention over 4 weeks–12 weeks,^21^ investigating their levels of CLA-producing gut microbiota mid- and long-term. It is already possible to change the composition of the microbiota through food in the short term,^151^ but the objective should be to maintain these changes in the microbiota composition,^21^ so that the anticarcinogenic effect of the CLA produced would be perdurable.

Future longitudinal studies must analyze whether there are differences in the anticancer effect from CLA produced in the human intestine, where this process naturally occurs, compared with that from the oral intake of already-produced CLA. Similarly, analysis of the efficiency of producing CLA metabolites by target bacteria in the human intestine, compared with CLA supplementation and the current microbiome-based precision nutrition approaches, is recommended.

Similarly, future studies should focus on the anticarcinogenic effect of each specific CLA metabolite and on extending our knowledge of CLA-producing bacteria and their complex interactions with the rest of the human microbiota.

As knowledge of the CLA metabolites and the production pathways increases, the interest of society in CLA’s role in CRC is likely to increase. Consequently, research projects regarding its efficacy and safety in pre-clinical and human trials are needed,^38^ enabling complementing of current anticarcinogenic strategies with microbiome-based precision nutrition approaches.

## CONCLUSIONS

The CLA metabolite has an anticancer effect in CRC. CLA isomers may act against CRC by a number of different action mechanisms. Moreover, CLA isomers can be produced by several different bacterial pathways: directly from LA to CLA, indirectly through forming intermediate products, or by using VA as substrate. Various strategies have been developed to increase the amount and bioavailability of CLA in humans, but have important limitations: the intake of the precursor LA is not recommended for increasing the amount of CLA; CLA in supplements and enriched foods must pass through the entire gastrointestinal tract; and the effect of CLA-producing probiotic supplements usually lasts only while the consumption is maintained.

In this context, next-generation microbiome-based precision nutrition interventions constitute a promising strategy for overcoming these difficulties. Our review of the scientific evidence regarding bacteria naturally present in the human gut shows that at least 55 bacteria have shown a significant association with CLA production. After discarding those bacteria associated with detrimental or inconsistent effects, 38 show strong evidence of CLA production. Therefore, according to the current scientific evidence, we can affirm that the human gut microbiota has the potential to produce CLA endogenously.

However, important issues need resolving before applying this knowledge in practical precision nutrition: (i) are the CLA metabolites produced by the human gut equally anticarcinogenic? (ii) will an increase in the amount of CLA-producing bacteria correlate with an increase in the amount of bioavailable CLA? (iii) can future precision nutrition approaches increase the amount of CLA-producing bacteria enough? and (iv) could this hypothetical increase in CLA-producing bacteria negatively impact the overall equilibrium of the gut microbiota?

This review has revealed new research lines that could be useful for increasing our understanding of CLA and its promising application in a next-generation microbiome-based nutrition-precision CRC therapeutic tool.
